# Iterative Data-adaptive Autoregressive (IDAR) whitening procedure for long and short TR fMRI

**DOI:** 10.3389/fnins.2024.1381722

**Published:** 2024-08-02

**Authors:** Kun Yue, Jason Webster, Thomas Grabowski, Ali Shojaie, Hesamoddin Jahanian

**Affiliations:** ^1^Department of Biostatistics, University of Washington, Seattle, WA, United States; ^2^Department of Radiology, University of Washington, Seattle, WA, United States; ^3^Department of Neurology, University of Washington, Seattle, WA, United States

**Keywords:** serial correlation, pre-whitening, autoregressive model, short-TR fMRI, resting-state fMRI, task fMRI, type-I error, temporal resolution

## Abstract

**Introduction:**

Functional magnetic resonance imaging (fMRI) has become a fundamental tool for studying brain function. However, the presence of serial correlations in fMRI data complicates data analysis, violates the statistical assumptions of analyses methods, and can lead to incorrect conclusions in fMRI studies.

**Methods:**

In this paper, we show that conventional whitening procedures designed for data with longer repetition times (TRs) (>2 s) are inadequate for the increasing use of short-TR fMRI data. Furthermore, we comprehensively investigate the shortcomings of existing whitening methods and introduce an iterative whitening approach named “IDAR” (Iterative Data-adaptive Autoregressive model) to address these shortcomings. IDAR employs high-order autoregressive (AR) models with flexible and data-driven orders, offering the capability to model complex serial correlation structures in both short-TR and long-TR fMRI datasets.

**Results:**

Conventional whitening methods, such as AR(1), ARMA(1,1), and higher-order AR, were effective in reducing serial correlation in long-TR data but were largely ineffective in even reducing serial correlation in short-TR data. In contrast, IDAR significantly outperformed conventional methods in addressing serial correlation, power, and Type-I error for both long-TR and especially short-TR data. However, IDAR could not simultaneously address residual correlations and inflated Type-I error effectively.

**Discussion:**

This study highlights the urgent need to address the problem of serial correlation in short-TR (< 1 s) fMRI data, which are increasingly used in the field. Although IDAR can address this issue for a wide range of applications and datasets, the complexity of short-TR data necessitates continued exploration and innovative approaches. These efforts are essential to simultaneously reduce serial correlations and control Type-I error rates without compromising analytical power.

## 1 Introduction

Most fMRI studies utilize the blood-oxygenation-level-dependent (BOLD) signal, which reflects the convolution of neuronal activity and cerebral hemodynamic response function (HRF) that persists for several seconds. This gives rise to temporally correlated data points within the obtained time-series data. In addition, physiological noise such as respiration and heart rate may also contribute to the serial correlations in observed fMRI signals. The existence of serial correlation among consecutive time points poses inherent challenges in the analysis of fMRI data. For example, the usual standard error of the sample correlation coefficient between two time series is biased, which could lead to incorrect conclusions in linear regression analysis (Woolrich et al., 2001; Afyouni et al., 2019). These challenges are particularly pertinent when using a generalized linear regression model, the most common method for analyzing fMRI data, to explore the relationship between fMRI observations and task regressors in task-fMRI studies. Eklund et al. (2016) have substantiated the considerable role of poorly modeled temporal autocorrelation in the surge of type-I errors. Additionally, existing models that describe brain functional connectivity networks (second-order structure), such as graphical models (Belilovsky et al., 2016; Monti et al., 2017) and generalized autoregressive conditional heteroscedastic (GARCH) time series models (Lindquist et al., 2014; Riccelli et al., 2017; Lee and Kim, 2021), are built upon the assumption of independence among time points, implying the absence of any serial correlations within the observations. In addition, serial correlations can lead to biased estimations and distort the computation of variances (Arbabshirani et al., 2014).

To address this issue, whitening procedures are often employed to remove serial correlations in fMRI data. Whitening often involves estimating the serial correlations from the data and using the inverse correlation matrix to de-correlate the observations. There have been extensive discussions regarding the appropriate approaches for whitening (Woolrich et al., 2001; Lenoski et al., 2008; Bright et al., 2017; Corbin et al., 2018; Olszowy et al., 2019; Luo et al., 2020). Among model-based approaches, the autoregressive model (AR) and autoregressive moving average (ARMA) model with fixed order has been a standard practice for whitening time series observations in popular fMRI software packages. Examples include Analysis of Functional NeuroImaging (AFNI) (Cox, 1996) using a voxel-specific ARMA(1,1) model (Chen et al., 2012), and Statistical Parametric Mapping (SPM) (Penny et al., 2011) as well as FMRIB Software Library (FSL) (Jenkinson et al., 2012) using a global AR(1) model (Friston et al., 2002). However, most of these traditional approaches were developed for fMRI data acquired with longer repetition times (TRs) of ~2 s. Recent advancements in imaging technology, however, have enabled the acquisition of fMRI measurements with much shorter TRs ( ≤ 0.5 s), resulting in higher levels of serial correlations with slower decay rates per time-point. Traditional whitening procedures often misrepresent serial correlation for short-TR observations, primarily due to the utilization of low model order, such as AR(1) or AR(1,1), as well as the assumption of uniform AR coefficients across the brain (Sahib et al., 2016; Bollmann et al., 2018; Luo et al., 2020). As a result, there is a pressing need for better modeling approaches that account for temporal correlations in fMRI time series, especially given the increasing adoption of short-TR fMRI data as the standard in the field. Recent studies suggest that using higher-order AR models with spatially-varying coefficients can effectively whiten the time series, even when dealing with sub-second TRs (Sahib et al., 2016; Bollmann et al., 2018; Luo et al., 2020). Sahib et al. (2016) explored the effect of high-order AR models (up to an order of 20) on increasing the T-values in task-fMRI studies using FMRISTAT (Worsley et al., 2002). Luo et al. (2020) proposed data-adaptive high-order AR(*p*) model with order *p* selected based on Akaike information criterion (AICc). However, as we will demonstrate in this study, these methods may still remain insufficient to remove the serial correlations in short-TR datasets. Specifically, over 88% of the nodes have significant remaining serial correlations after whitening with a high-order AR model (Section 3). This poses big challenges for subsequent analysis employing models like graphical models and GARCH models, which rely on the assumption of independent observations. Furthermore, as will be illustrated in Section 3, different whitening approaches can yield significant variations in resulting signals, subsequently influencing downstream analyses and conclusions. This underscores the pressing concern of replicability within fMRI research. Therefore, it is crucial to develop universally adaptable whitening approaches that are capable of accommodating diverse fMRI dataset characteristics.

To address the limitations of current whitening procedures, we improve the currently available high-order AR model-based whitening models, and propose an iterative whitening procedure, named *IDAR* (for Iterative Data-adaptive AR). IDAR is built on high-order AR models, and is featured with its iterative nature and a more flexible and data-adaptive order. While the single iteration version of IDAR (referred to as *IDAR-iter1* in the rest of the paper, where we set max.iteration to 1) essentially covers the high-order AR models proposed in Luo et al. (2020) and Sahib et al. (2016), the iterative nature of IDAR enables it to more effectively model the complicated serial correlation structure in short-TR fMRI, which is often not adequately captured by a single AR model. Specifically, in the empirical studies, while IDAR-iter1 leaves 88% of the nodes with remaining serial correlations, IDAR successfully reduces the percentage to < 1% (Section 3). The proposed IDAR approach is applicable to both short-TR and long-TR fMRI datasets. In addition to assessing the residual serial correlations post-whitening, we also explore the impact of whitening on type-I errors in simulated task-based fMRI using obtained resting-state fMRI data. We conduct a comparison of IDAR against the most widely-used prewhitening methods currently available, including the AR(1) model (as implemented in FSL and SPM), the AR(1,1) model (utilized by AFNI), and high-order AR models (represented by IDAR-iter1), using short-TR and long-TR datasets. Remarkably, prior studies have not simultaneously addressed both key facets of whitening procedure performance: the removal of serial correlation in resting-state fMRI analyses and the control of type-I errors in task-based fMRI experiments, making our investigation comprehensive in nature.

Our proposed IDAR offers users the flexibility to focus either on eliminating serial correlations or reducing type-I errors, tailored to the temporal resolution of their data and specific analytical needs. This adaptability empowers users to tailor their preprocessing to align with their particular objectives, effectively addressing the challenges associated with short-TR fMRI data.

## 2 Method

**Figure 1:**
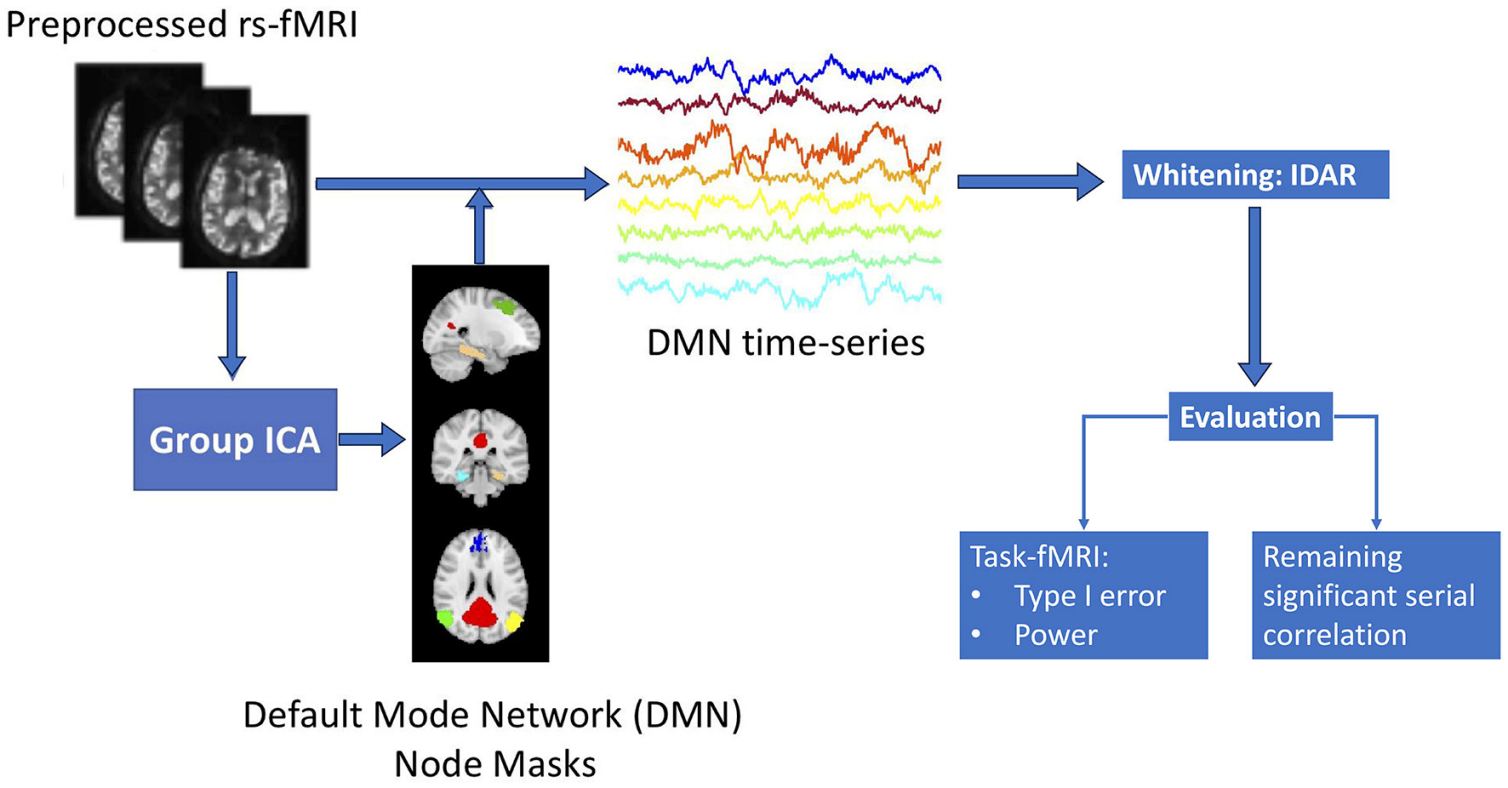
Flowchart of the structure for this study.

Figure 1 provides a summary of the sequential analysis steps employed in this study. Initially, the collected resting-state fMRI data undergoes standard preprocessing. To manage the processing load of our analyses more efficiently, we focus on the default mode network (DMN), which is distributed throughout the brain and offers a representative view of the spatial variations of serial correlation across the brain. We use group independent component analysis (ICA) to obtain brain masks for the eight nodes in DMN, based on which we obtain time series observations for each of the DMN brain nodes. Subsequently, we apply the proposed IDAR model to the node-wise time series observations for whitening. To assess the efficacy of the whitening algorithm, we evaluate it from two perspectives: (1) the extent of residual serial correlations after whitening, which is crucial for enabling subsequent analysis assuming independent observations, and (2) the impact of whitening on power and type-I error within the context of simulated task-fMRI using resting-state fMRI data.

### 2.1 Data

We employed two data sets, one with long TR at 2.5 s and the other with short TR at 0.49 s. This study was approved by the Internal Review Board (IRB) of Stanford University (short-TR data) and the University of Washington (long-TR data) and was Health Insurance Portability and Accountability Act (HIPAA) compliant. This study adhered to the highest ethical standards and was conducted in compliance with the principles outlined in the Declaration of Helsinki. Informed consent was obtained from all participants after a comprehensive explanation of the study's purpose, procedures, potential risks, and benefits. The confidentiality and privacy of participants are strictly maintained throughout the research process. Internal review boards approved the consent procedure.

#### 2.1.1 Long TR dataset

The long TR dataset consisted of *n* = 87 subjects. We conducted all MRI data acquisitions on a research-dedicated 3T Philips Achieva scanner equipped with a 32-channel receiver coil. During the scanning session, participants were instructed to lie in the scanner with their eyes open while wearing a Pearl-Tec^®^ Crania to minimize head motion.

For registration and volumetric measurements, 3D T1 data were acquired from all participants using a conventional MPRAGE sequence with the following parameters: spatial resolution = 1 × 1 × 1mm^3^, 150 slices, flip angle = 8°, TR/TE = 8.8/4.6 ms, SENSE acceleration factor = 2, and matrix size = 256 × 256 × 176.

Resting-state functional data were collected using axial whole-brain multi-echo T2-weighted acquisition with a scan duration of 10 min, using 3.5 mm isotropic voxels and TR/TEs = 2,500/9.5, 27.5, 45.5 ms. To ensure data quality, we performed motion correction using FSL's MCFLIRT and removed non-brain matter with the FSL Brain Extraction Tool (Jenkinson et al., 2012). Subsequently, we processed the multi-echo BOLD data using AFNI's specialized module TEDANA, designed for multi-echo planar imaging and analysis with independent component analysis (ME-ICA). TEDANA allows for the differentiation between BOLD (neuronal) and non-BOLD (artifact) components by leveraging the characteristic linear echo-time dependence of BOLD T2 signals (Kundu et al., 2012). This approach produced recombined images optimally weighted across the three echo times, along with ME-ICA-denoised time series. Following this, the data were co-registered to T1 images and normalized to the Montreal Neurological Institute template (MNI152 standard space) using FSL. We applied high-pass temporal filtering and regressed out the global signal and movement parameters estimated using FSL across six dimensions (lateral, vertical, and horizontal translation, and yaw, pitch, and roll rotation) to further enhance data quality.

#### 2.1.2 Short TR dataset

Data were collected from a cohort of 99 subjects using a 3T GE Discovery MR750 scanner equipped with a 32-channel head coil (NOVA Medical). Resting-state fMRI data were acquired utilizing a Simultaneous Multi Slice (SMS) EPI with blipped controlled aliasing in parallel imaging (CAIPI) sequence (Setsompop et al., 2012). The imaging parameters were set as follows: TR/TE = 490/30 ms, multiband acceleration factor of 5, CAIPI shift of FOV/3, field of view (FOV) = 24 × 24 cm^2^, voxel size = 3.14 × 3.14 × 4mm, and scan duration of 10 min (Jahanian et al., 2019). Prior to the functional scans, a high-order shimming procedure was implemented to reduce B0 inhomogeneity (Kim et al., 2002). The SMS EPI images were reconstructed using the SENSE/GRAPPA combination method (Blaimer et al., 2006). K-space calibration data were designed to correspond to a one-dimensional undersampling along the phase encoding direction, with an empirically set interpolation kernel of 7 × 4 (readout × phase encoding) acquired points for each missing point.

A high-resolution 3D T1-weighted image was acquired before the resting-state fMRI scans for spatial normalization and anatomical reference, utilizing an IR-SPGR sequence with the following parameters: TR/TE/TI = 8.18/3.2/900 ms, matrix = 256 × 256, in-plane resolution = 0.94 × 0.94 mm, and slice thickness/slice spacing = 1/0 mm, covering 176 sagittal slices.

The acquired resting-state fMRI images underwent preprocessing and analysis using various tools from FSL, AFNI, and custom MATLAB code. After discarding the first six volumes to ensure magnetic stabilization, the images were motion-corrected using least square minimization and normalized to the MNI152 standard space using the subject's high-resolution T1-weighted image with an affine linear registration technique involving 12 degrees of freedom (Jenkinson et al., 2002). To remove low-frequency signal drifts (< 0.01 Hz) from the data, a high-pass filter was applied. Additionally, two band-stop temporal filters targeting respiratory ([0.25–0.35] Hz) and cardiac ([0.8–1.02] Hz) frequency bands were applied using 5th order Butterworth IIR filters (Jahanian et al., 2019). Various sources of variance including movement (Lund et al., 2005), cerebrospinal fluid, white matter (Dagli et al., 1999; Weissenbacher et al., 2009), and global signal (Desjardins et al., 2001; Greicius et al., 2003) were removed using multiple linear regression. Variance associated with movement parameters was estimated using FSL across six dimensions (lateral, vertical, and horizontal translation, and yaw, pitch, and roll rotation). Cerebrospinal fluid and white matter confounds were calculated from a 3 mm spherical ROI placed in the ventricles and the white matter, respectively.

### 2.2 Whitening algorithm

The proposed IDAR for whitening fMRI observations is an iterative and data-driven whitening model based on high-order AR models. The basic concept involves estimating serial correlations through AR models, with the selection of AR orders guided by the corrected Akaike Information Criterion (AICc) (Hurvich and Tsai, 1989). The iterative procedure is motivated by the fact that persistent residual serial correlations are frequently observed when attempting to de-correlate the data using a single AR model, even when a high-order AR model is used. This phenomenon is particularly evident when processing datasets with short TRs. This observation suggests that the serial correlation structure may not be adequately explained by a single AR model. We thus adopt an iterative approach that estimates any remaining serial correlations of the whitened observations from the preceding iteration. The whitening procedure is described in Algorithm 1 for a univariate time series *Y*(*t*) with *T* total observations.

**Algorithm 1:**
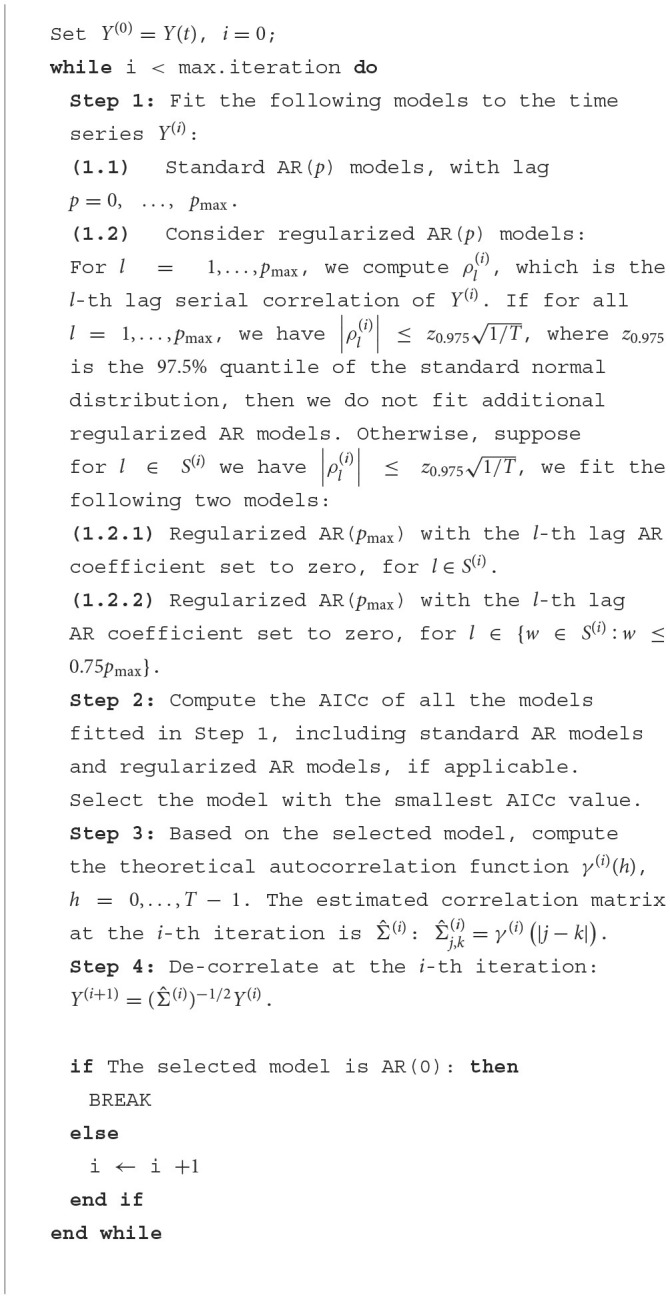
Algorithm for whitening based on IDAR.

When the algorithm stops at iteration *K*, the resulting whitened time series, *Y*^(*K*)^, has the corresponding estimated serial correlation matrix $\prod_{i=1}^{K}{\left({\hat{\Sigma }}^{\left(K-i+1\right)}\right)}^{-1/2}{\left(\prod_{i=1}^{K}{\left({\hat{\Sigma }}^{\left(K-i+1\right)}\right)}^{-1/2}\right)}^{⊤}$. We find that setting the maximum number of iterations to 5 is typically sufficient, even for data sets with short TRs. In selecting the best model in each iteration, information criteria such as AIC and Bayesian Information Criterion (BIC) can be employed. For this study, we use AICc due to its relatively superior capability in detecting the appropriate AR orders (Sen et al., 2002). Through our investigation, we determine that setting the maximum lag order as *p*_max_ = 10/TR achieves satisfactory performance. This choice assumes that the correlations significantly diminish beyond 10 s, which reasonably aligns with the time duration of typical hemodynamic response functions (Friston et al., 2000; Buxton et al., 2004; Handwerker et al., 2004; Arichi et al., 2012). When dealing with a short TR and a large number of observations per time series, fitting all *p*_max_ models in Step (1.1) would be time-consuming. As a practical solution, we employ the step-wise selection approach (auto.arima) implemented in the R package forecast (Hyndman et al., 2023). In this approach, we initialize the lag values at *p*_max_, *p*_max_/2, and *p*_max_/4. The inclusion of regularized models in Step (1.2) is motivated by observations made while processing datasets with short TRs. When the TR is small, fitting a full AR(*p*_max_) model can be both time-consuming and at risk of overfitting. The utilization of regularized models addresses these concerns while still effectively targeting high serial correlations. Specifically, the regularized model employed in Step (1.2.2) incorporates additional higher-order AR lags to ensure the accurate capture of high-order serial correlations.

We have observed that the estimated correlation matrices, ${\hat{\Sigma }}^{\left(i\right)}$, can occasionally have extremely large condition numbers, leading to unstable numerical performance. This is likely due to fitting high-order AR models to a limited number of observations and the resulting autocorrelation matrices are rank deficient. To address this issue, we regularize the estimated correlation matrix using a banding approach (Bickel and Levina, 2008). Specifically, we progressively set the last 10 non-zero autocorrelations in ${\hat{\Sigma }}^{\left(i\right)}$ to zero until the condition number falls below 1 × 10^8^: we will first set ${\hat{\Sigma }}_{j,k}^{\left(i\right)}=0$ for |*j* − *k*| ≥ *T* − 10, and if the condition number is still above the threshold, continue to set ${\hat{\Sigma }}_{j,k}^{\left(i\right)}=0$ for |*j* − *k*| ≥ *T* − 20. Exploring other covariance regularization approaches falls outside the scope of this study; however, it would be an interesting direction for future research.

Our approach is distinctive in its iterative whitening of the data, stacking multiple AR models to estimate the serial correlation structure. While this necessitates the fitting of additional parameters, we have observed that a singular AR model is often inadequate for whitening, especially for short-TR data. Conversely, our algorithm automatically accounts for low-order AR models and single-iteration AR models, rendering it suitable for both short-TR and long-TR data analyses.

### 2.3 Evaluation approaches

We assess the efficacy of the IDAR-based whitening algorithm from two perspectives: (1) the degree of residual serial correlations after the whitening process, which is especially relevant to subsequent analysis of resting-state fMRI that assumes independent observations, and (2) the impact of whitening on power, type-I error and accuracy in the context of task-fMRI, simulated using resting-state fMRI data.

To evaluate the residual serial correlations, we adopt the evaluation procedure outlined in Corbin et al. (2018). After applying the whitening algorithm to a single time series, we utilize the Ljung-Box test (Ljung and Box, 1978) at lags ranging from 1 to 20/TR (Corbin et al., 2018). We control family-wise type-I error using Holm's (1979) method. We consider the time series not adequately whitened if the smallest adjusted *p*-value among the lags is < 0.05. We calculate the percentage of such inadequately whitened time series, where a successful whitening procedure should yield < 5% of inadequately whitened time series.

**Figure 2:**
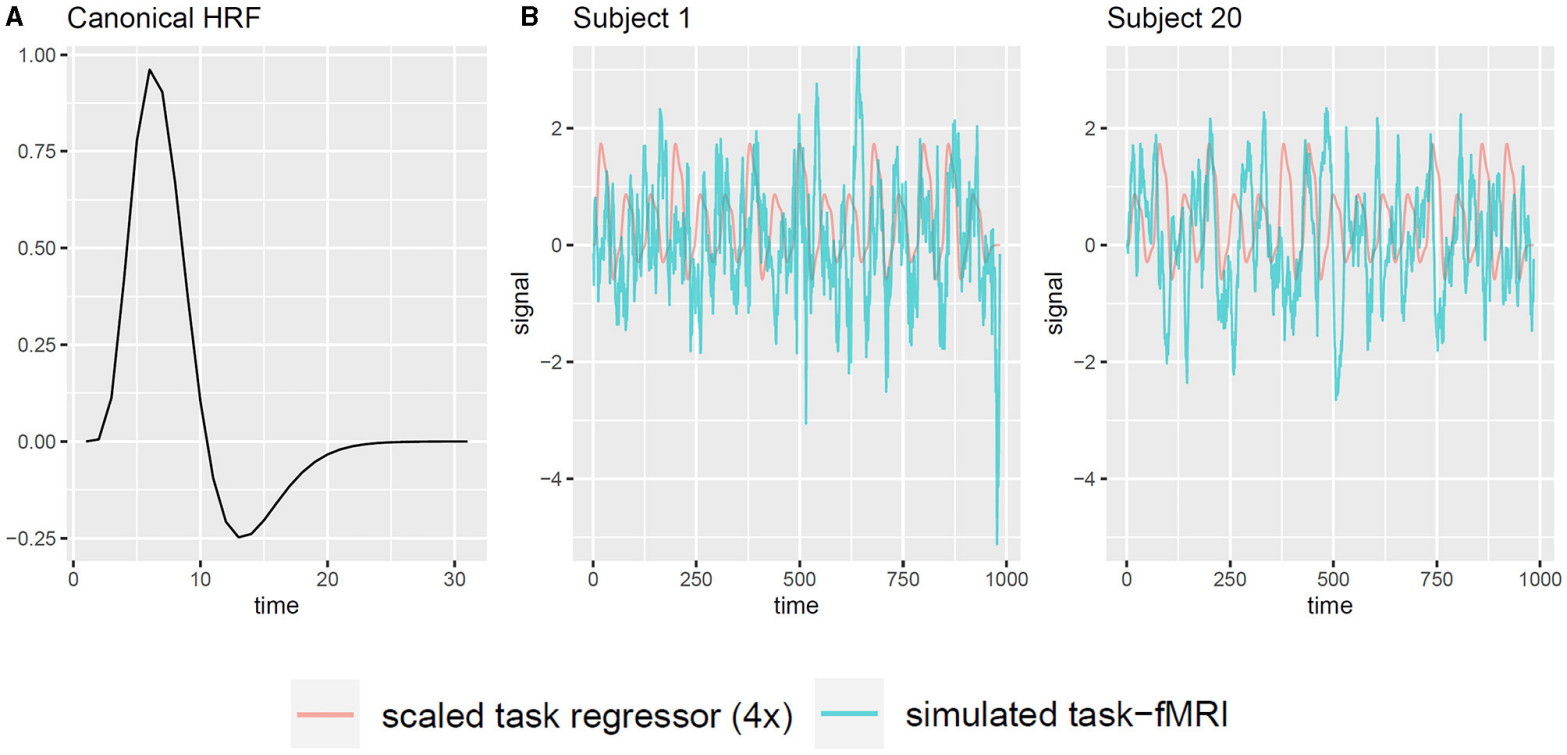
Illustration of generating task-fMRI signals from resting-state fMRI observations. **(A)** The canonical HRF used to convolve with the task paradigm. **(B)** Two example subjects' simulated task regressor and the corresponding simulated task-fMRI for node PCC.

Additionally, we examine the applicability of the whitening procedure to task-fMRI that analyzes the association between fMRI signals and event paradigms. In such analyses, the presence of serial correlation in the observations diminishes the effective sample size, which potentially leads to inflated type-I error rates in association tests based on the generalized linear regression model (GLM) (Penny et al., 2011; Luo et al., 2020). Therefore, we investigate whether the proposed whitening algorithm effectively controls the type-I error rate. Moreover, we assess whether whitening has any adverse effect on the statistical power and accuracy. Since assessing type-I error and power requires the knowledge of true signal, we use simple simulation studies based on the available resting-state fMRI data. For each subject, we simulate a single task regressor, denoted as *X*, based on a “boxcar” task paradigm: the time course is divided into blocks of 30 s, consisting of a 15-s task epoch followed by a 15-s resting epoch. Each task epoch is randomly assigned as either task A or task B with equal probability, with task A having twice the intensity level of task B. We convolve the boxcar task paradigm with the canonical hemodynamic response function (HRF) to obtain the unscaled task regressor, using the R function hrfConvolve from R package FIAR (v0.6, Roelstraete and Rosseel, 2011) with default double gamma function parameters. Although each brain region may exhibit slightly different HRFs in reality, our separate experiments revealed that small variations in HRF had minimal impact on the results. Thus, for the sake of simplicity, we adopt a static default HRF across all brain nodes. Figure 2A illustrates the canonical HRF used in the simulation. Considering a signal-to-noise (SNR) ratio of 0.1, we scale the task regressor such that its mean value equals one-tenth of the standard deviation of the raw resting-state fMRI data (Welvaert and Rosseel, 2013), which yields a contrast-to-noise ratio from 0.33 to 0.55 among all subjects. Note that here we choose SNR = 0.1 for clarity. Our separate analyses confirmed that varying SNR levels did not alter the conclusions drawn in this study. Consequently, the simulated task-fMRI data, denoted as *Y*_*ts*_, are obtained by adding the scaled task regressor *X* to the raw resting-state fMRI data *Y*_*rs*_ (Figure 2B). Next, we use the following simple linear model to analyze the association between the fMRI signal *Y* and the simulated task regressor *X*:


$$\begin{array}{l} Y=\beta_{0}+\beta_{1}X+\epsilon , \end{array}$$

where β_0_, β_1_ ∈ ℝ are the regression coefficients, and ϵ is the error term. In our analysis, we use the simulated task-fMRI data *Y* = *Y*_ts_ as the outcome variable to examine the power of detecting the association between task-fMRI and the task regressor. The power is quantified as the proportion of brain nodes exhibiting a significant association between the simulated task-fMRI and the task regressor across all subjects. Additionally, for the same set of brain nodes, we employ their resting-state fMRI data *Y* = *Y*_rs_ as the null outcome to investigate the type-I error. Here, type-I error is defined as the proportion of brain nodes displaying a significant association between the resting-state fMRI data and the simulated task regressor across all subjects. Furthermore, we compute an accuracy metric that integrates findings from both type-I error and power analyses. We use a Wald statistic to test the hypothesis *H*_0_:β_1_ = 0, where β_1_ represents the association between the task regressor *X* and the outcome *Y*. The outcome *Y* will be whitened before fitting the linear regression model, and the regressors will be multiplied with the de-correlation matrix accordingly. Specifically, suppose the whitening procedure estimates a data correlation matrix $\hat{\Sigma }$, we essentially fit the following model to estimate the association between ${\hat{\Sigma }}^{-1/2}Y$ and ${\hat{\Sigma }}^{-1/2}X$:


$$\begin{array}{l} {\hat{\Sigma }}^{-1/2}Y=\beta_{0}{\hat{\Sigma }}^{-1/2}+\beta_{1}{\hat{\Sigma }}^{-1/2}X+{\hat{\Sigma }}^{-1/2}\epsilon . \end{array}$$

In addition to evaluating the proposed IDAR-based whitening procedure, we include standard whitening procedures based on AR(1) model and the ARMA(1,1) models for comparison. Moreover, we investigate the performance of IDAR-iter1, which essentially encompasses the high-order AR models proposed in Luo et al. (2020) and Sahib et al. (2016). This comparison provides valuable insights into the performance of existing whitening approaches in the context of both long-TR and short-TR datasets. We also include results obtained from the non-whitened outcome for comparison.

## 3 Results

**Figure 3:**
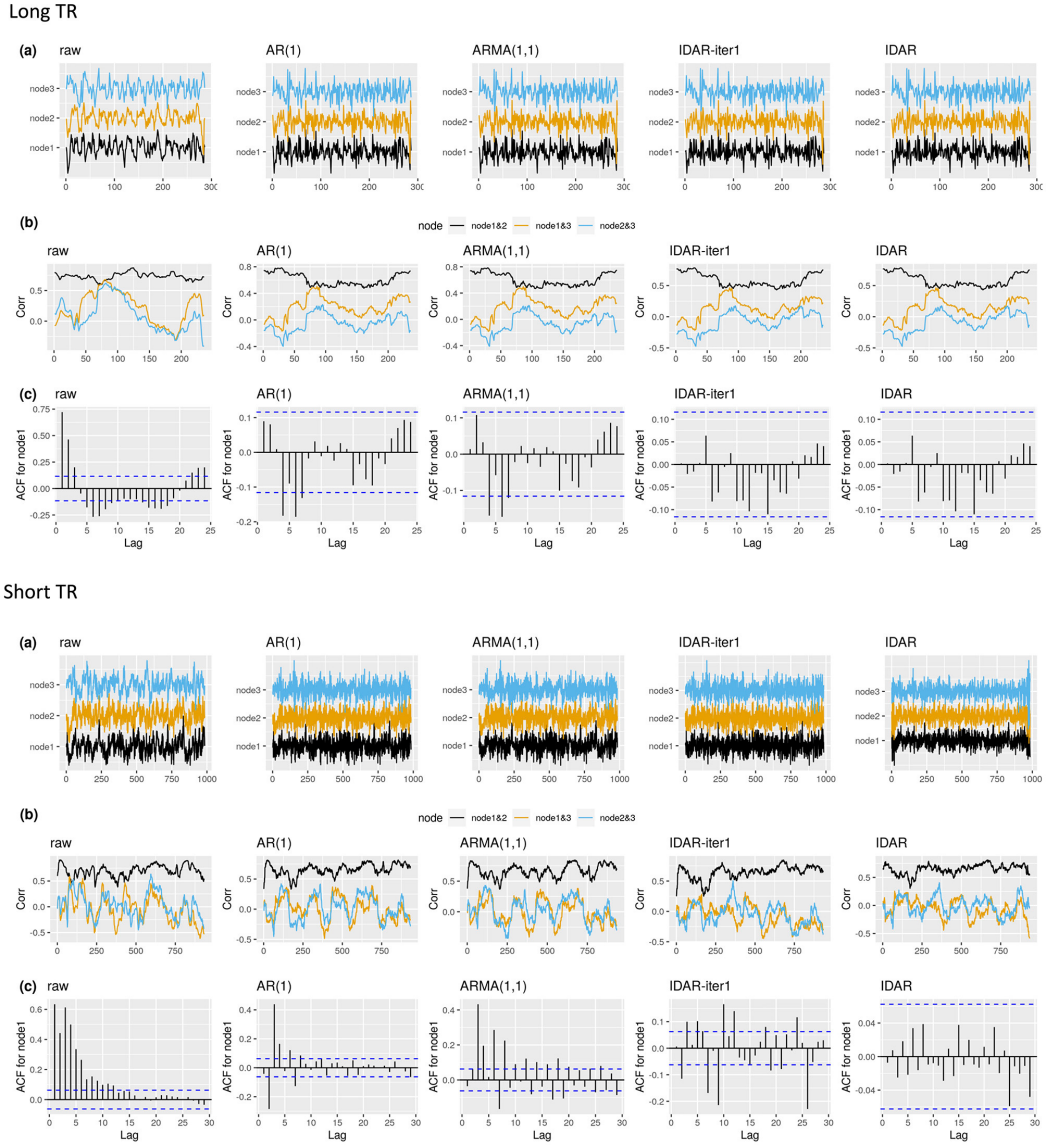
The resting-state fMRI signal with different whitening procedures for two example subjects. Top panel shows an example subject from long-TR dataset; bottom panel shows an example subject from short-TR dataset. We select to present the signals from node 1—SFG left, node 2—SFG right, node 3—MPFC. We show the raw resting-state fMRI, as well as the whitened signals after applying the IDAR, IDAR-iter1, AR(1) and ARMA(1,1) based whitening procedures. **(A)** The raw and whitened resting-state fMRI observations. **(B)** The sliding-window-based correlation profile, computed from raw and whitened observations. A window length of 100s is selected for illustration purpose. **(C)** The serial correlations at different lags for observations from node 1. The dashed lines represent the threshold, beyond which the serial correlation is considered significantly different from zero at 0.05 significance level.

We begin by illustrating the raw and whitened resting-state fMRI signals of an example subject (Figure 3). For clarity, we only showcase data from three nodes within the DMN. The application of various whitening techniques did not significantly alter the resting-state fMRI profiles, with the resulting signals demonstrating visual consistency across the different whitening methods (Figure 3A). For a simple and non-rigorous visualization of the correlation profiles, we show the sliding-window-based correlations based on the raw signals and whitened signals (Figure 3B). We notice changes in the sliding-window-based correlation profile after applying whitening procedures to the raw signal. Nevertheless, these differences remain visually inconspicuous across the various whitening methods, as depicted in Figure 3B. The serial correlations unveil distinct patterns. Specifically, the raw signals were highly serially correlated, while the application of standard whitening methods such as AR(1) and ARMA(1,1) resulted in the persistence of relatively large serial correlations at lag 4 and lag 6 for node 1 (Figure 3C). Meanwhile, the IDAR-based whitening approaches successfully removed serial correlations in the selected signals.

**Figure 4:**
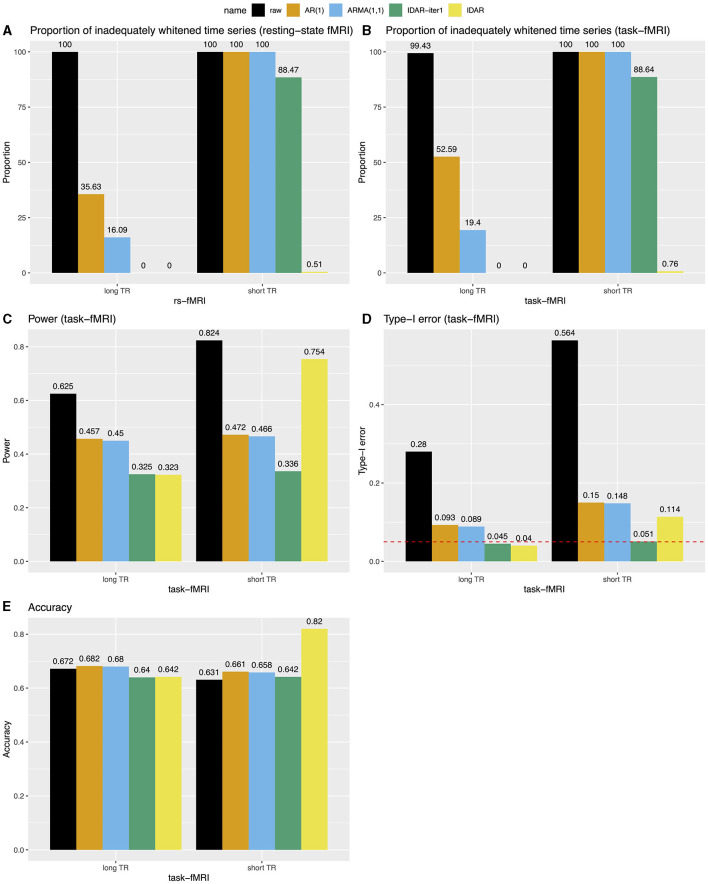
Bar charts for comparing the performance of different whitening approaches in long-TR and short-TR datasets. **(A, B)** Proportion of time series that are not adequately whitened for resting-state fMRI and task-fMRI. **(C)** Power for detecting the association between signals and task paradigm in task-fMRI analysis. **(D)** Type-I error for testing the association between signals and task paradigm in task-fMRI analysis. The red dashed line represents the 0.05 nominal level. **(E)** Accuracy for testing the association between signals and the task paradigm in task-fMRI analysis, combining test results from both type-I error and power analyses.

Figure 4 provides a comprehensive comparison of the performance of various whitening algorithms. The proposed IDAR approach effectively removes serial correlations in nearly all analyzed time series, irrespective of whether short-TR or long-TR datasets were analyzed, or whether resting-state fMRI or simulated task-fMRI signals were considered. In instances of long-TR datasets, where serial correlations diminish rapidly, the IDAR-iter1 strategy proficiently removes serial correlations. However, this method falls short in addressing the short-TR datasets, as a considerable 88% of the provided time series still display significant residual serial correlations. In contrast, conventional procedures based on AR(1) or ARMA(1,1) models proved ineffective in achieving optimal whitening, particularly in the context of short-TR datasets. Specifically, with the long-TR dataset, at least 16.09% of time series did not attain adequate whitening, while with the short-TR dataset, none of the time series achieved satisfactory whitening (Figures 4A,B).

We subsequently investigated the power, type-I error and accuracy concerning simulated task-fMRI data analysis. We observed a decrease in the statistical power of testing the associations between the task paradigm and the fMRI signal following the application of the whitening procedures (Figure 4C). Figure 4D presents the corresponding type-I error rates. In the absence of any whitening procedure, the type-I error could reach as high as 0.56. Whitening procedures lead to a substantial decrease in type-I error. Notably, the IDAR-iter1 method effectively maintained the type-I error at the nominal level for both long-TR and short-TR datasets, whereas the IDAR approach demonstrated an inflated type-I error of 0.11 within the short-TR dataset. The conventional AR(1) and ARMA(1,1) approaches exhibit inflated type-I error rates across both datasets. In terms of accuracy, IDAR demonstrated the highest performance for the short-TR dataset, while the accuracies across different whitening approaches were comparable for the long-TR dataset.

## 4 Discussion

Whitening plays a pivotal role in addressing serial correlations in fMRI data and stands as a critical pre-processing step. Conventional whitening techniques prove to be inadequate for fMRI data collected with short TR. Furthermore, as highlighted in Section 3, the utilization of various whitening methods can introduce substantial disparities in the resulting signals, consequently impacting subsequent analyses and conclusions. This underscores the issue of replicability in the field of fMRI research and the need for one unified whitening approach that accommodates the diverse characteristics inherent in fMRI datasets.

In response to the challenge of whitening fMRI signals, we introduced a novel iterative data-driven whitening procedure, termed IDAR. This approach offers versatility by effectively managing serial correlations for subsequent analysis necessitating independent observations in resting-state fMRI studies, while also controlling the type-I error rate in task-based fMRI analyses. IDAR is grounded in high-order AR models, with IDAR-iter1 encompassing existing high-order AR model-based whitening techniques. What sets IDAR apart is its iterative nature, surpassing conventional high-order AR models in its capacity to capture the complex serial correlation structures in fMRI datasets. Initially developed for ROI-level analysis, IDAR is directly applicable to voxel-level signals. We have conducted separate voxel-level analyses and confirmed that our conclusions remain consistent when applied to voxel-level signals (see Appendix A for details).

Our focus is primarily on whitening approaches that utilize time series models to estimate serial correlations. Notably, no existing method satisfactorily addresses both residual serial correlations and type-I error in task analysis simultaneously. For datasets with longer TR, standard approaches employing low-order AR models have been extensively studied. However, these models, such as AR(1) and ARMA(1,1), often lack the requisite order to adequately capture serial correlation structures, leading to incomplete removal of serial correlations. These standard models, widely used in popular fMRI analysis softwares such as AFNI, FSL and SPM, encounter further challenges in the context of short-TR datasets due to the presence of slow-decaying serial correlations, as our results in Section 3 confirm. Although prior literature has suggested high-order AR models as effective for sub-second TR datasets (Sahib et al., 2016; Bollmann et al., 2018; Luo et al., 2020), our findings in Section 3 reveal that even the flexible high-order AR model (represented by IDAR-iter1) struggles with short-TR datasets. Such unaddressed serial correlations can introduce biases in estimation, distort variance computations, and pose challenges in justifying subsequent analyses involving models that assume independent observations, such as graphical models and GARCH models.

We also investigated the performance of the whitening procedures in the context of task-fMRI analysis using simulated task-fMRI data. The standard low-order models showed inflated type-I error rates that lead to false discovery, potentially impacting clinical applications like brain tumor surgery or epilepsy treatment (Orringer et al., 2012). Interestingly, while the high-order AR model (IDAR-iter1) could not fully mitigate serial correlations, it demonstrated a capacity to control type-I error at the nominal level for both short-TR and long-TR datasets, at the expense of statistical power. Although the IDAR approach with multiple iterations fell short of fully controlling type-I error for short-TR datasets, it provided substantially higher statistical power compared with other methods. Thus, complexities arise when comparing the performance of whitening procedures across different evaluation metrics. Notably, the evaluation of whitening procedures lacks a consensus on proper metrics, and our chosen criteria are not exhaustive. Our analysis reveals instances where methods excel in one metric but fall short in another. The relationship between these metrics remains unclear, underscoring the need for exploring appropriate evaluation measures for whitening techniques. Such endeavors could significantly contribute to advancing the field.

Our proposed IDAR approach has demonstrated superior efficacy in removing the serial correlation in both long-TR and short-TR datasets, compared with currently available methods. In the context of long-TR data, the adaptive order-selection mechanism inherent in IDAR encompasses low-order models automatically. Notably, IDAR-iter1 is often sufficient for long-TR signals, and this aligns with the prevailing understanding that long-TR datasets typically exhibit fast-decaying serial correlations, making moderate-order time series models usually sufficient. Conversely, for short-TR datasets, the IDAR approach is very efficient in removing the serial-correlation from the data where the existing methods are not effective. This is attributed to its incorporation of high-order AR models and its capacity to address intricate serial correlation structures through iterative procedures.

Leveraging more comprehensive models, IDAR has shown improved control over type-I error rates compared to traditional whitening procedures. However, in the context of long-TR task-fMRI analysis, IDAR and higher-order AR models (equivalent to IDAR-iter1) tend to exhibit reduced statistical power. In contrast, during short-TR task-fMRI analysis, IDAR outperforms both AR(1) and ARMA(1,1) models in terms of statistical power and control over type-I error and was significantly superior to higher-order AR models in terms of statistical power. Optimizing statistical power while ensuring strict control of type-I error rates is a promising direction for future research in this field.

Although IDAR provided significant improvement over conventional methods in terms of addressing serial correlation, power and type-I error, for both short-TR and long-TR data, neither IDAR nor IDAR-iter1 simultaneously addresses residual correlations and inflated type-I error. Researchers can leverage IDAR-iter1 to address the elevated type-I error rates in task-fMRI analysis of short-TR data, while IDAR may be more suitable for various other applications and datasets. Nonetheless, the ongoing challenge is to create methods that successfully strike a balance between completely whitening short-TR datasets and retaining their analytical power. The complexities of short-TR data necessitate continued exploration and innovative approaches to simultaneously reduce serial correlations and control type-I error rates, without compromising analytical power. Whether an optimal model can be achieved by adjusting IDAR model parameters or necessitates an entirely different model framework remains uncertain. Moreover, the optimal model parameters may vary from dataset to dataset due to the complexity of fMRI datasets, presenting an interesting direction for future research.

The IDAR algorithm may become computationally intensive when analyzing voxel-wise signals from short-TR datasets due to the need for fitting restricted high-order AR models to a large number of observations. Future research aimed at enhancing the computational efficiency of the IDAR algorithm would make it more practical for use across different datasets and for voxel-wise signal analysis.

## Data availability statement

The raw data supporting the conclusions of this article will be made available by the authors, without undue reservation.

## Ethics statement

This study was approved by the Internal Review Board (IRB) of Stanford University (short-TR data) and the University of Washington (long-TR data) and was Health Insurance Portability and Accountability Act (HIPAA) compliant. The studies were conducted in accordance with the local legislation and institutional requirements. The participants provided their written informed consent to participate in this study.

## Author contributions

KY: Conceptualization, Data curation, Formal analysis, Investigation, Methodology, Validation, Writing – original draft, Writing – review & editing, Software, Visualization. JW: Data curation, Formal analysis, Visualization, Writing – original draft, Writing – review & editing. TG: Data curation, Funding acquisition, Writing – original draft, Writing – review & editing. AS: Funding acquisition, Investigation, Methodology, Resources, Supervision, Validation, Writing – original draft, Writing – review & editing. HJ: Conceptualization, Data curation, Formal analysis, Funding acquisition, Investigation, Methodology, Resources, Supervision, Validation, Writing – original draft, Writing – review & editing.
