# Supplementary material for: Iterative Data-adaptive Autoregressive (IDAR) whitening procedure for long and short TR fMRI
Source: Front Neurosci. 2024 Aug 2;18:1381722. doi: 10.3389/fnins.2024.1381722 (PMC11327036; doi:10.3389/fnins.2024.1381722)
Supplement: Supplementary file 1 [file Image_1.pdf]

Supplement: Iterative Data-adaptive Autoregressive (IDAR) whitening procedure for long and short TR fMRI

Kun Yue[1], Jason Webster[2], Thomas Grabowski[2,3], Ali Shojaie[1,*], and Hesamoddin Jahanian[2,*]

[1]Department of Biostatistics, University of Washington, Seattle

[2]Department of Radiology, University of Washington, Seattle

[3]Department of Neurology, University of Washington, Seattle

[*]Corresponding authors: Ali Shojaie, ashojaie@uw.edu; Hesamoddin Jahanian, hesamj@uw.edu

To illustrate the performance of the proposed IDAR algorithm, we conducted additional analyses using voxel-wise signals for a few representative subjects from both the long-TR and short-TR datasets. We employed the same simulation settings and analytical procedures as described in the main paper, with the sole modification being the utilization of voxel-level signals from the entire brain volume, rather than the DMN node-level signals. Figure S1 and Figure S2 present the evaluation metrics for selected subjects from the long-TR and short-TR datasets, respectively. These metrics exhibit consistent behavior with the results shown in Section 3, specifically in Figure 4.

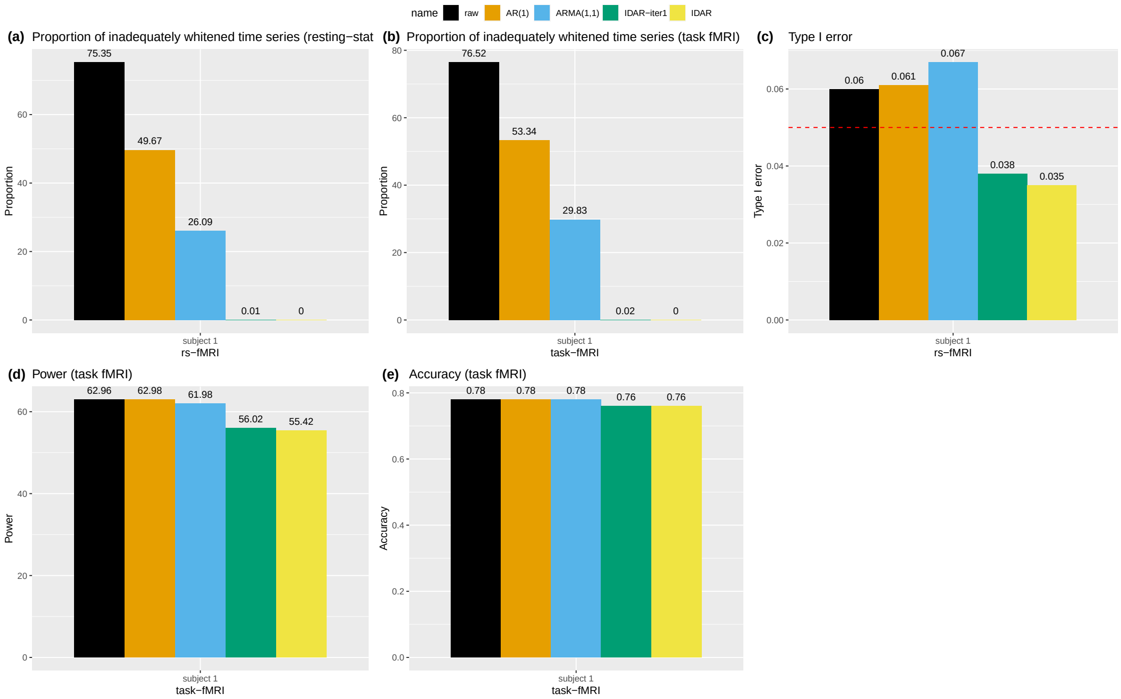

Fig. S1: Evaluation metrics for one example subject with long-TR signals.

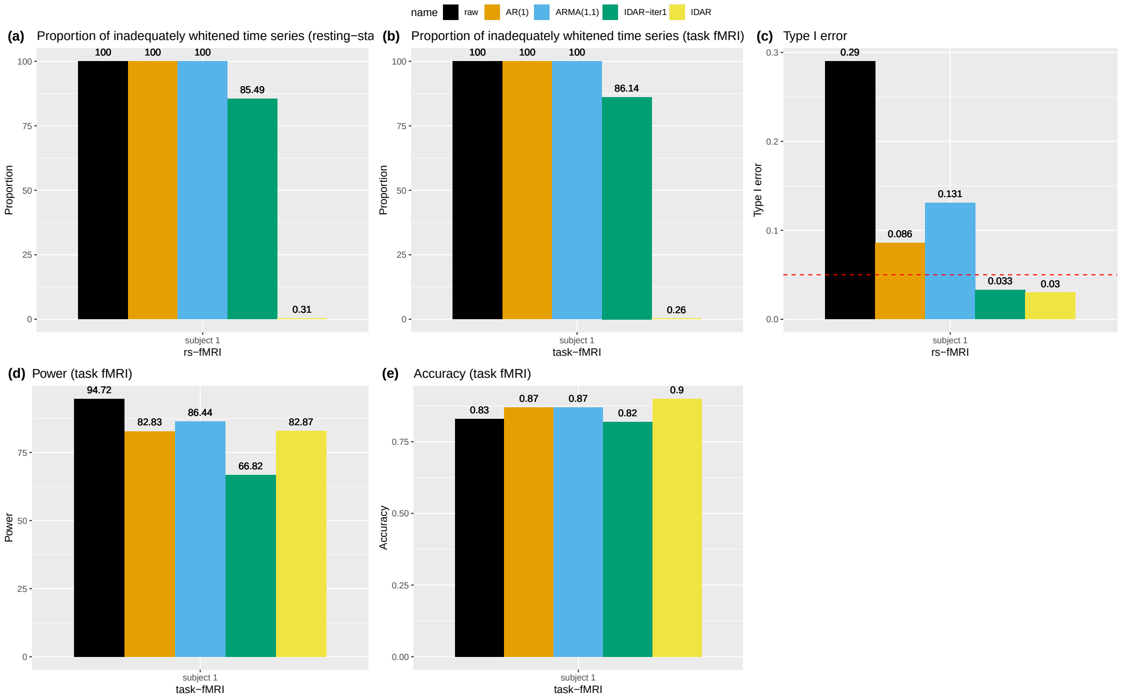

Fig. S2: Evaluation metrics for one example subject with short-TR signals.

Additionally, we plotted the brain maps of the evaluation metrics on a representative brain slice for the example subjects. The metric maps are displayed in Figures S3 through S5 for the long-TR dataset example, and in Figures S6 through S8 for the short-TR dataset example.

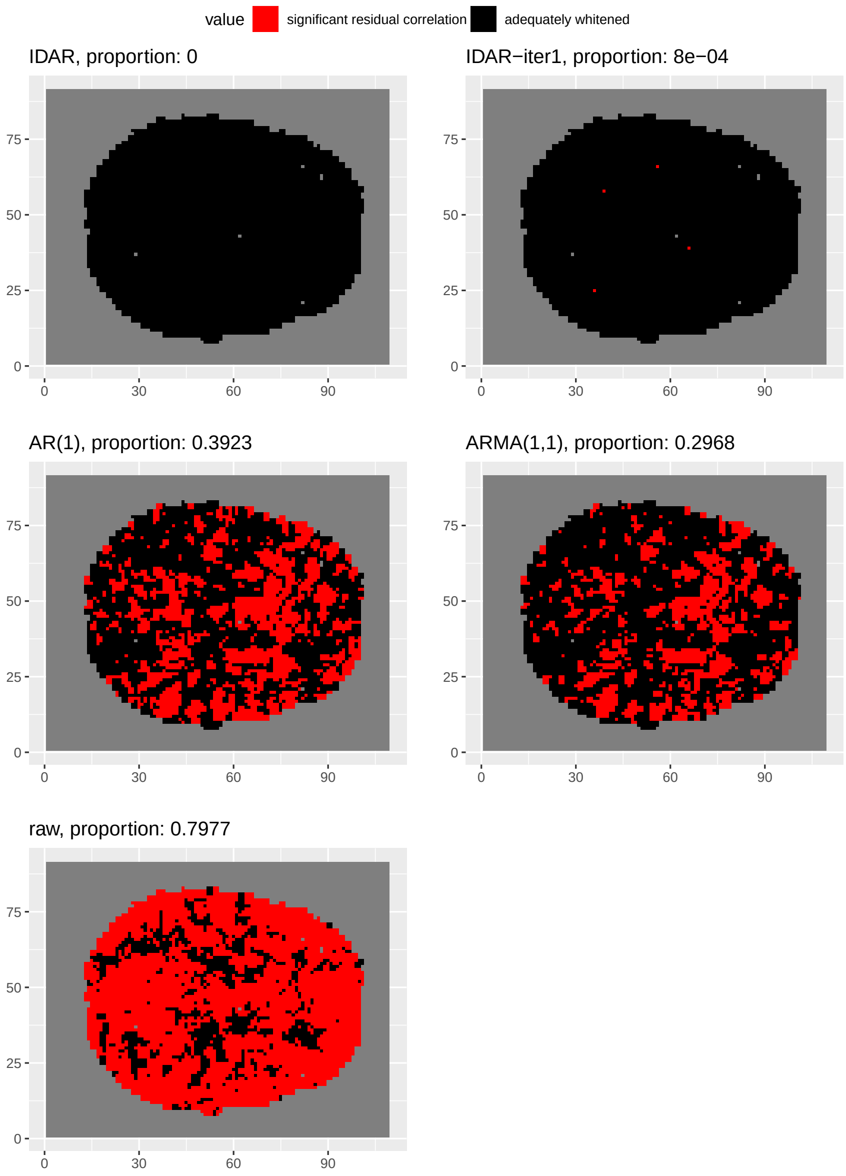

Fig. S3: Brain map of significant residual serial correlations. We present an example brain slice from a random subject of the long TR dataset. Voxels with significant serial correlations are highlighted in red.

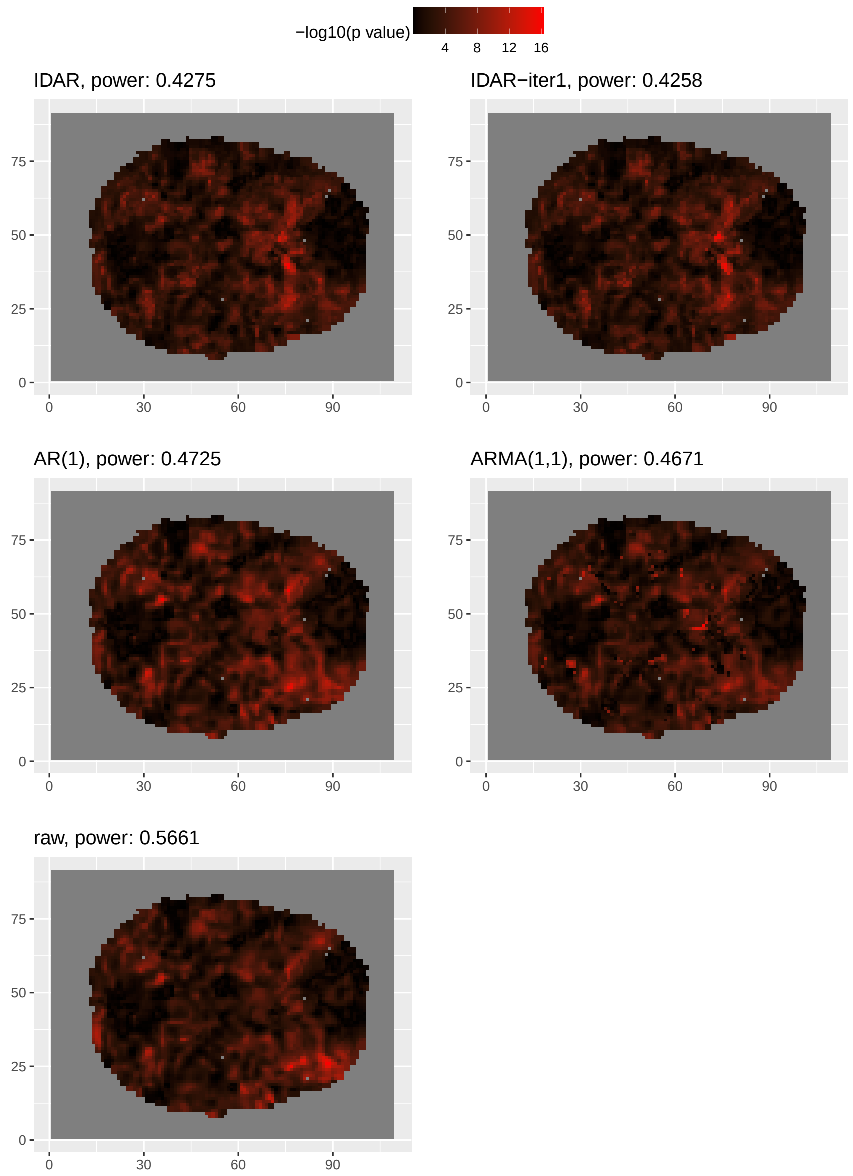

Fig. S4: Brain map of (transformed) p values in task-fMRI analysis. We present an example brain slice from a random subject of the long TR dataset.

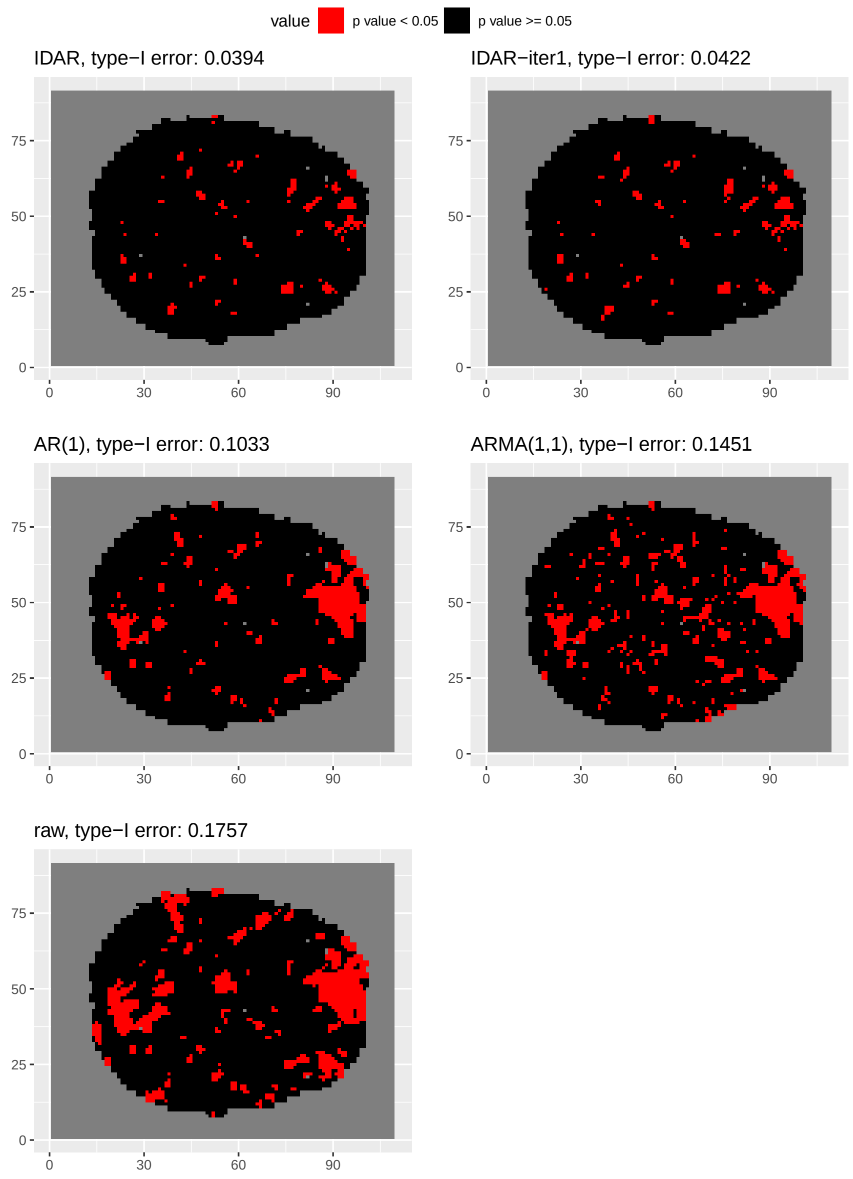

Fig. S5: Brain map of type-I error. We present an example brain slice from a random subject of the long TR dataset. Voxels with type-I error are highlighted in red.

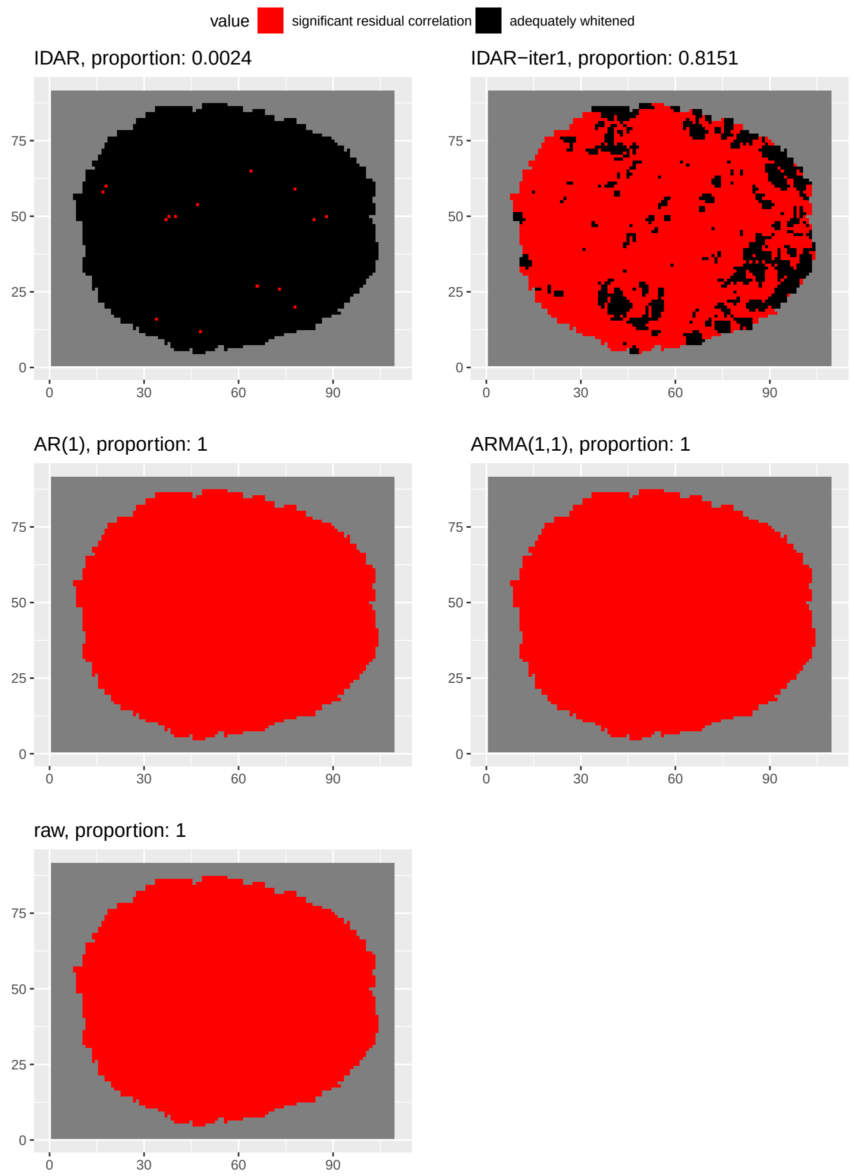

Fig. S6: Brain map of significant residual serial correlations. We present an example brain slice from a random subject of the short TR dataset. Voxels with significant serial correlations are highlighted in red.

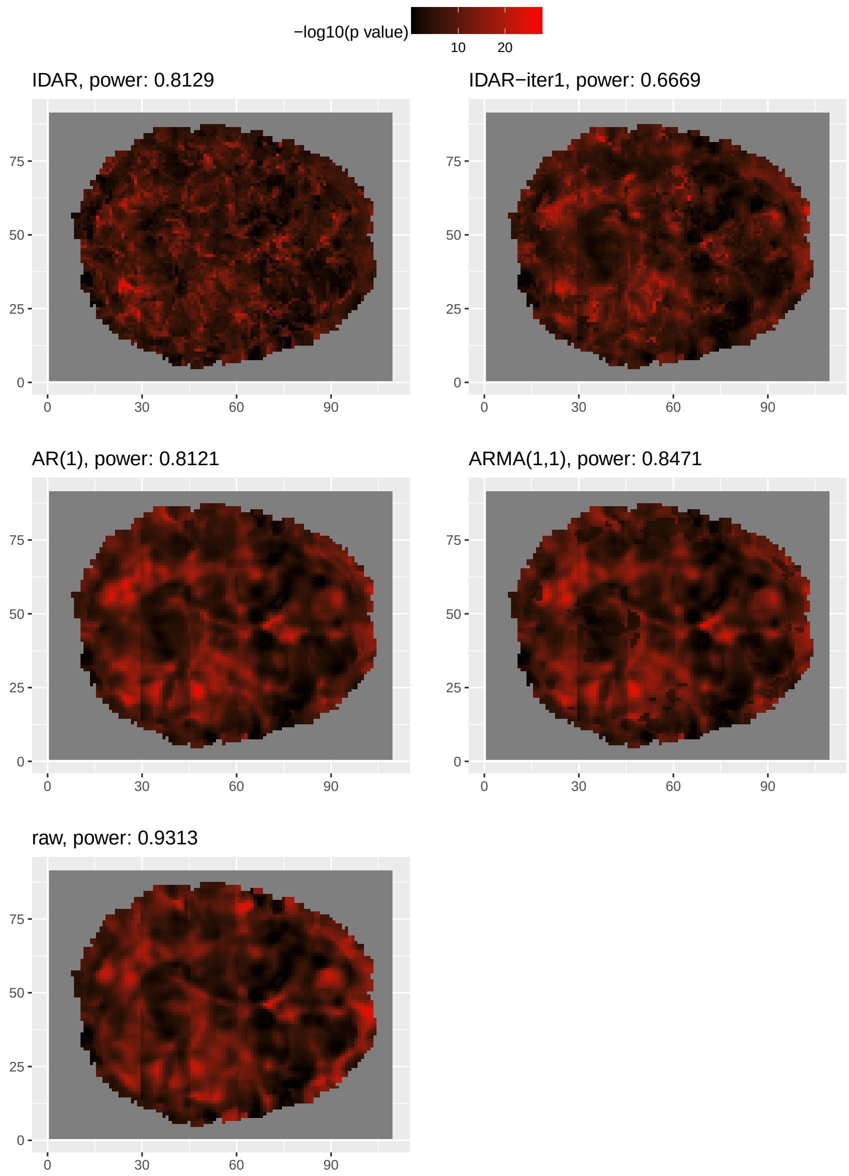

Fig. S7: Brain map of (transformed) p values in task-fMRI analysis. We present an example brain slice from a random subject of the short TR dataset.

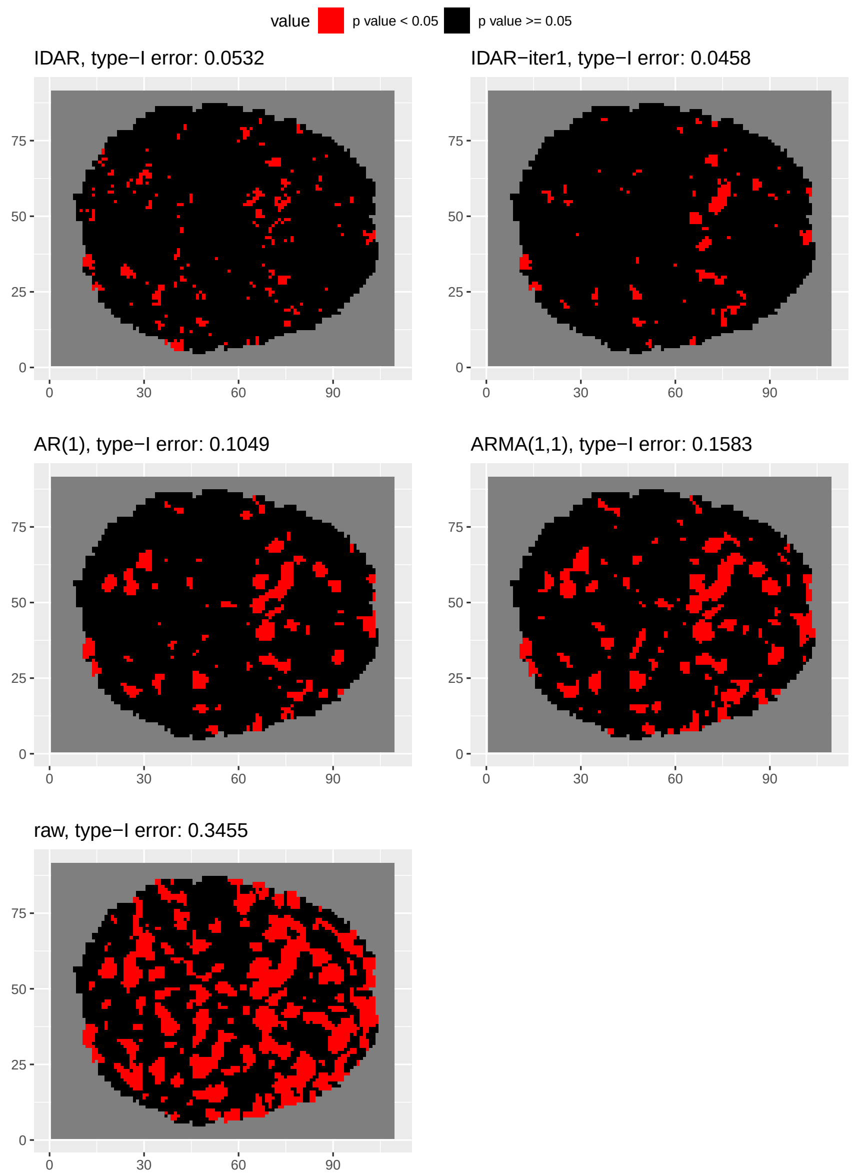

Fig. S8: Brain map of type-I error. We present an example brain slice from a random subject of the short TR dataset. Voxels with type-I error are highlighted in red.
